# Total knee arthroplasty following previous hardware implantation: do hardware removal strategies influence periprosthetic joint infections? A systematic review and meta-analysis

**DOI:** 10.1530/EOR-24-0100

**Published:** 2025-02-03

**Authors:** Domenico De Mauro, Chiara Comisi, Enrico Festa, Tiziana Ascione, Massimo Mariconda, Giovanni Balato

**Affiliations:** ^1^ Orthopedics and Traumatology Unit, Department of Public Health, “Federico II” University, Naples, Italy; ^2^ Department of Orthopedics and Rheumatological Sciences, Fondazione Policlinico Universitario A. Gemelli IRCCS, Rome, Italy; ^3^ Department of Orthopedics and Geriatric Sciences, Catholic University of the Sacred Heart, Rome, Italy; ^4^ Service of Infectious Diseases, AORN Antonio Cardarelli Hospital, Naples, Italy

**Keywords:** total knee arthroplasty, hardware removal, periprosthetic joint, infection, conversion TKA

## Abstract

**Purpose:**

- Total knee arthroplasty (TKA) in patients who underwent previous knee surgeries can be a challenging procedure both technically and for the complication rate. Conversion TKA is affected by a higher risk of infection compared to primary TKA. The aims of this meta-analysis are i) to compare the infectious risk among patients undergoing TKA after a prior hardware implantation, evaluating removal vs maintenance of the hardware, and ii) within the removal group, to compare staged vs concurrent procedure.

**Methods:**

- In accordance with the PRISMA guidelines, a systematic literature review was conducted up to January 2024. The review was registered in the PROSPERO database: CRD42024510444. The inclusion criteria comprised the following: i) patients aged 18 years or older, ii) individuals who had undergone total knee replacement and iii) those with a history of prior nonabsorbable hardware implantation. The pooled incidence of periprosthetic joint infections (PJI) was reported using odds ratios with corresponding 95% confidence intervals (CIs).

**Results:**

- The investigation of database and references identified 284 studies. PJI risks differed significantly among groups, with a higher risk in the removal group (*z* = 3.5630, *P* = 0.0004). Furthermore, within the removal group, the risk of PJI was lower in cases of staged removal compared to concurrent removal (*z* = 2.0931, *P* = 0.0363).

**Conclusions:**

- TKA following a previous knee hardware implantation indicates a higher PJI risk when the hardware is removed compared to leaving it in place. If hardware removal is necessary, staged removal is recommended. The presence of minor hardware is the only scenario where, if removal is necessary, one-stage approach is preferred.

## Introduction

Total knee arthroplasty (TKA) has proven to be a successful, safe and cost-effective treatment for advanced knee osteoarthritis (1). The incidence of TKA has steadily increased over the past 25 years across all ages (2), and its percentage is expected to increase by 143% until 2050 (3).

Post-traumatic arthritis of the knee is the third most common cause of total knee replacement after primary arthritis and rheumatoid arthritis (4, 5). Distal femoral and proximal tibial surgery is relatively common in young patients as a result of high-energy trauma or congenital deformities. Commonly, previously performed surgeries, including open reduction and internal fixation (ORIF), intramedullary nailing (IMN), high tibial osteotomy (HTO) or distal femur osteotomy (DFO) and ligamentous reconstruction, involve hardware implantation, which may directly or indirectly affect future arthroplasty procedure (6, 7). Conversion TKA (cTKA), defined as TKA performed in patients with retained or removed hardware, can lead to excellent outcomes but may represent a clinical and technical challenge (8). Indeed, studies about cTKA in patients who underwent previous knee surgeries have demonstrated longer operative times, increased blood losses, higher readmission rates, increased number of mechanical complications, higher revision rates and especially high infection risk (9, 10, 11). The hardware management, including removal and the choice of the optimal time (concurrent or staged) to mitigate the related complications, is poorly described. In particular, several questions remain unanswered, such as whether an actual difference in infection rate exists between removed and retained hardware and between staged and concurrent hardware removal. Moreover, the optimal time to wait after hardware removal to proceed with cTKA is still controversial.

Therefore, this meta-analysis aims to compare the infectious risk among patients undergoing TKA after a prior hardware implantation, specifically evaluating outcomes associated with the removal vs maintenance of the existing hardware; to assess and compare infectious risks in patients undergoing a staged procedure vs those undergoing a concurrent procedure involving both hardware removal and TKA; and to analyze the infectious risk based on the type of hardware (minor vs major) to determine the most effective removal strategies.

## Materials and methods

### Search strategy and eligibility criteria

In accordance with the Preferred Reporting Items for Systematic Reviews and Meta-Analyses (PRISMA) guidelines (12), a systematic review of the literature was conducted up to January 2024. This systematic review focused on the examination of clinical outcomes and complication rates in patients undergoing TKA after hardware implantation. The search strategy included three online databases: MEDLINE, Web of Science and Scopus. The keywords used for the research were combined as follows: ‘TKA’ or ‘TKR’ or ‘total knee arthroplasty’ or ‘total knee replacement’ or ‘conversion TKA’ and ‘previous osteosynthesis’ or ‘hardware removal’ or ‘hardware’ or ‘plate’ or ‘screw’ not ‘periprosthetic fracture’ and relative MeSH combinations.

The inclusion criteria were not limited to English language literature and specific publication dates. Reference lists of the selected articles were searched for additional papers that were not identified in the database search.

To avoid overlap with other ongoing review studies, the protocol was registered online with the International Prospective Register of Systematic Reviews (PROSPERO) before starting the review.

Longitudinal studies, both retrospective and prospective, along with randomized controlled trials, were subjected to thorough evaluation and subsequently included in the final reference list.

The inclusion criteria comprised the following: i) patients aged 18 years or older, ii) individuals who had undergone total knee replacement and iii) those with a history of prior nonabsorbable hardware implantation, including osteosynthesis for fractures, anterior cruciate ligament (ACL) reconstruction and tibial or femoral osteotomy.

The exclusion criteria were as follows: i) case reports, expert opinions, previous systematic reviews and letters to the editor, ii) studies that did not assess infectious complications in outcomes analysis and iii) studies with incomplete data and those for which corresponding authors did not respond to our data request via mail.

### Study assessment and data extraction

Initially, the titles and abstracts of the studies underwent screening by two independent reviewers (EF and CC). Full texts were obtained for all abstracts that appeared to meet the inclusion criteria or presented any uncertainty. Subsequently, each study was analyzed based on the inclusion criteria by two independent reviewers (DDM and TA), and any discrepancies in inclusion were resolved through assessment by the senior author (GB). Relevant data were systematically extracted from each study, including participant demographics, sample size, hardware type and quantities, surgical details and outcomes and complications.

A comparative analysis was conducted based on the declared endpoints, involving a comparison of infectious outcomes between hardware removal and hardware maintenance, a comparison of infectious outcomes between concurrent and staged removal and a comparison of infectious outcomes between minor or major surgeries, based on the type of hardware used. In particular, plates and screws, along with intramedullary nails, were categorized as major hardware, while staples, buttons, rods and wires were classified as minor hardware.

The methodological quality of the studies incorporated into this meta-analysis was evaluated using the methodological index for non-randomized studies (MINORS) score (13), which provides a maximum score of 16 and 24 for noncomparative and comparative studies, respectively. Two authors (TA and MM) independently determined the MINORS score, and the final score was derived through consensus.

### Statistical analysis

The analysis utilized the log odds ratio (OR) as the outcome measure. Given the anticipated heterogeneity among the included studies, a random-effects model was employed for data. The extent of heterogeneity (*τ*^2^) was estimated using the restricted maximum-likelihood estimator (14). In addition, the analysis reports the *Q*-test for heterogeneity and the *I*^2^ statistic (15). In the event of detecting any level of heterogeneity (*τ*^2^ > 0), a prediction interval for the true outcomes is also presented. Studentized residuals and Cook’s distances were employed to assess whether studies could be outliers and/or exert influence within the model framework. Studies with a studentized residual exceeding the $\left\{100\times \left[1-\frac{0.05}{2k}\right]\right\}$th percentile of a standard normal distribution were deemed potential outliers. This classification applied a Bonferroni correction, adopting a two-sided *α* = 0.05 significance level for studies included in the meta-analysis. Studies with a Cook’s distance exceeding the median plus six times the interquartile range of the Cook’s distances were identified as influential. Funnel plot asymmetry was assessed using both the rank correlation test and the regression test, employing the standard error of the observed outcomes as the predictor. The pooled incidence of PJI was reported using ORs with corresponding 95% confidence intervals (CIs). SPSS statistic software (IBM Corp. IBM SPSS Statistic for MacOS, Version 29.0. IBM Corp, 2023) was used for all statistical analyses. *P* ≤ 0.05 was considered significant.

## Results

### Search and selection process

**Figure 1:**
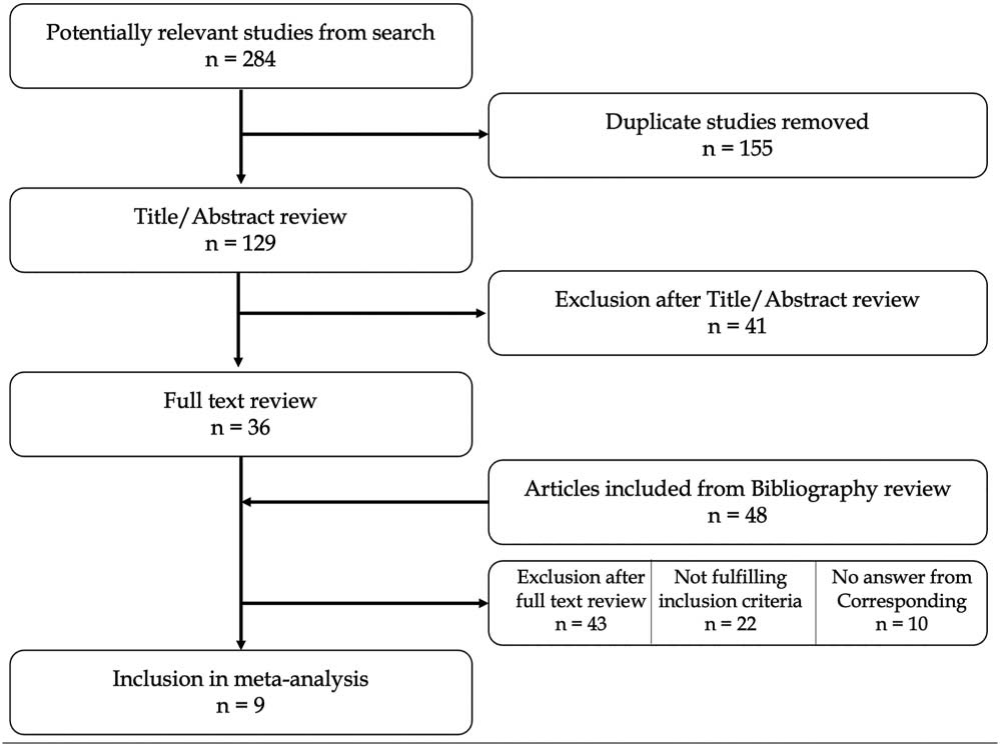
Study flow chart.

**Figure 2:**
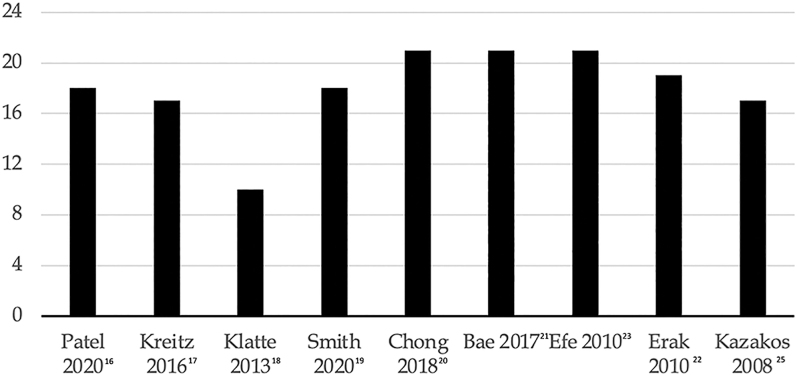
MINORS score.

The study flow chart is presented in Fig. 1. The initial literature search included 284 articles, with 155 duplicates subsequently removed. Following this initial step, the remaining 129 papers underwent screening based on titles and abstracts. After excluding papers in languages other than English, the full texts of 36 articles were further assessed for eligibility. Through this full-text analysis, an additional 48 articles were included in the review, sourced from references in the full-text papers admitted for analysis. Following the comprehensive search, papers not meeting inclusion criteria were excluded, along with those for which corresponding authors did not provide additional data after an official request by mail. Ultimately, 11 papers were included in this systematic review (8, 16, 17, 18, 19, 20, 21, 22, 23, 24, 25). Notably, two studies were excluded from the meta-analysis due to the absence of events reported for each arm, rendering it unsuitable for data pooling and incongruent with the meta-analytic strategy. The quality analysis of the included studies was assessed through MINORS score. The results are shown in Fig. 2.

### Descriptive data of included studies

Eleven studies were incorporated into the systematic review (8, 16, 17, 18, 19, 20, 21, 22, 23, 24, 25), covering a timeframe from 2008 to 2020. Altogether, 682 patients with a previous hardware implantation who underwent TKA were identified (Table 1).

**Table 1:** Characteristics of the studies included in this systematic review.

Study	Location	Study type	Patients, *n*	% Male	Age* (mean)	Previous knee surgery	Hardware type	Hardware site	Follow-up (months)*
Kazakos *et al.* (25)	Greece	RS	39	26	55.6	ACLR	Screws, staples	Femur, tibia	NR
Efe *et al.* (23)	Germany	RS	63	63	58.7	DS	Plates	Femur, tibia	NR
Erak *et al.* (22)	Canada	RS	124	53	61.9	DS	Screws, plates	Femur, tibia	64.8
Klatte *et al.* (18)	Germany	RS	155	81	66.5	DS	Screws, staples, plates, nail, buttons, rods	Femur, tibia, patella	43.6
Lin *et al.* (24)	Taiwan	RS	101	56	54.0	DS	Screws, staples, plates, nail, buttons, rods	Femur, tibia	10.4
Manrique *et al.* (8)	USA	RS	29	0	68.3	DS	NR	Femur, tibia	74.4
Kreitz *et al.* (17)	USA	RS	41	21	69.0	DS	Screws, staples, plates, nail, buttons, rods	Femur, tibia	82.0
Bae *et al.* (21)	South Korea	RS	35	NR	57.0	HTO	Screws, plates	Tibia	NR
Chong *et al.* (20)	USA	RS	32	8	67.0	HTO	Staples, screws	Femur, tibia	54.0
Smith *et al.* (19)	USA	RS	8	4	65.0	DS	Screws, plates, nails	Femur, tibia, patella	NR
Patel *et al.* (16)	USA	RS	55	34	56.0	ACLR	Screws, staples	Femur, tibia	NR

NR, not reported; NA, not applicable; ACLR, anterior cruciate ligament reconstruction; HTO, high tibial osteotomy; and DS, different surgeries.

*Indicates mean values.

The mean age across the studies ranged from 54 to 69 years, with the weighted mean age of 61.7 ± 5.6 years. Of the 647 patients, 45% were male (range 4–81%) and the mean BMI was 30.9 ± 2.2. Based on the 11 included articles (8, 16, 17, 18, 19, 20, 21, 22, 23, 24, 25), the most common surgeries before primary TKA were ACL reconstruction with 140 patients (16, 20) and HTO with 137 patients (21, 22, 23, 25). Five studies (8, 17, 18, 19, 24), including 405 patients, described different types of surgical procedures with various hardware implantations, encompassing ORIF, IMN, HTO or DFO and ligamentous reconstruction. The anatomical site was described for all patients evaluated. Indeed, 327 patients were included in studies involving femur and tibia (16, 17, 18, 20), 210 were part of studies involving also the patella (8, 19), and finally, 137 patients were included in tibia-only studies (21, 22, 23, 25).

The weighted mean follow-up was 54.9 ± 25.8 years (range 1.5–5.9 years), and the mean time from previous surgery to TKA was 92.9 ± 27.7 months (Table 1).

The study by Kreitz *et al*. (17) was the only study reporting the diagnostic criteria used for infection, the Musculoskeletal Infection Society (MSIS). Furthermore, no data were provided about the pathogen population in different studies, except for Klatte a*et al*. (18), who specifically identified the most common isolated germ, *Staphylococcus aureus*.

### Removal vs maintenance: meta-analysis

**Figure 3:**
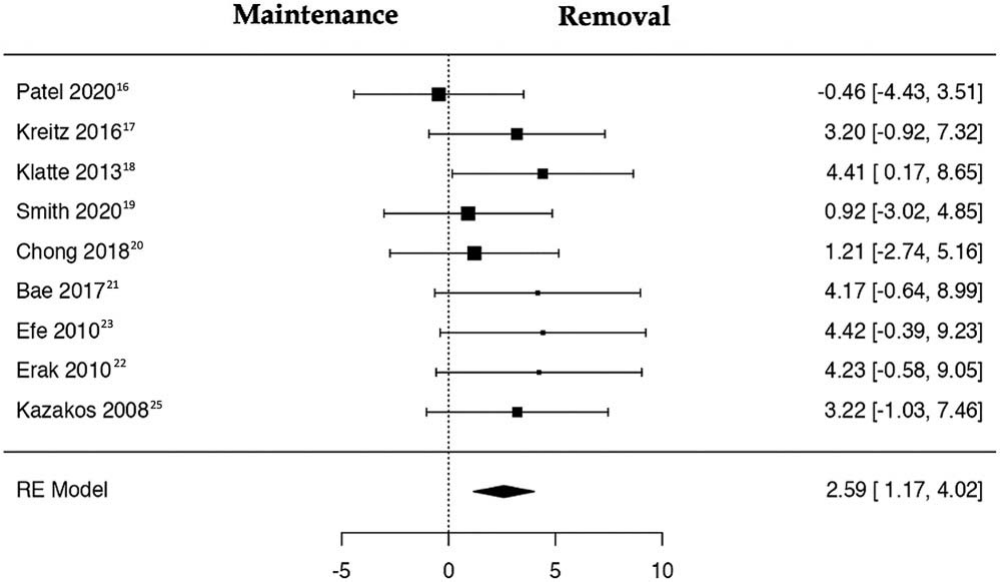
Risks of PJI in patients with previous hardware before total knee arthroplasty who underwent hardware removal vs those who did not undergo removal are depicted in the forest plot. The presented calculations were derived using random-effects models, and the confidence intervals are represented by the bars in the graph.

A total of nine studies (16, 17, 18, 19, 20, 21, 22, 23, 25) were included in the analysis, pooling data from 611 observations (patients with or without hardware removal before TKA) and identifying 48 events (postoperative PJI after cTKA). Utilizing the random-effects model, the data synthesis demonstrated a noteworthy elevation in the risk of PJI within the hardware removal group (log OR: 2.59, 95% CI: 1.17, 4.02), exhibiting a significant deviation from zero (*z* = 3.563, *P* = 0.0004) (Table 2). A positive OR indicates that the experimental group (removal) has a higher risk compared to the control group (maintenance). The relative forest plot is shown in Fig. 3.

**Table 2:** Meta-analysis results indicate a higher risk of infections in both removal vs maintenance (line 1) and staged vs concurrent removal (line 2) interventions. In both cases, the intervention groups (removal and staged) exhibit a greater risk of infections in TKA compared to the respective comparison groups, and these differences are statistically significant with a meaningful *P* value. In the sub-analysis of minor and major hardware, the results were significant for the comparison between staged and concurrent removal of minor implants (line 3) but not for major implants (line 4). Bold text indicates a significantly lower risk of infections in the comparison.

Implants	Intervention	Comparison	log OR	95% CI	*z*	*P* value
All	Removal	**Maintenance**	2.59	1.17, 4.02	3.563	0.0004*
All	**Staged**	Concurrent	1.83	0.15, 3.54	2.093	0.0363*
Minor	Staged	**Concurrent**	2.60	0.36, 4.84	2.274	0.0233*
Major	Staged	Concurrent	−0.70	−1.49, 0.09	1.748	0.0804

OR, odds ratio; CI, confidence interval.

*Statistically significant at *P* value <0.05.

**Figure 4:**
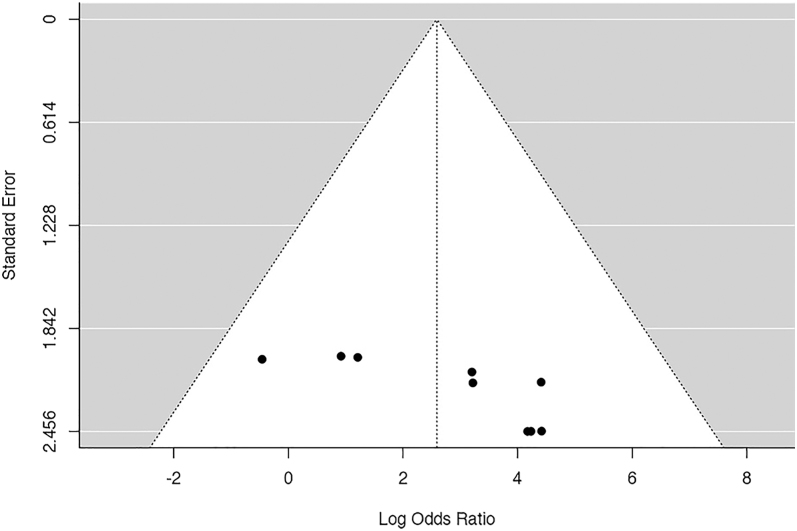
Funnel plot of the meta-analysis of maintenance vs removal: none of the studies could be considered excessively influential. Neither the rank correlation nor the regression test revealed any asymmetry in the funnel plot.

The *Q*-test results suggested a lack of significant heterogeneity in the true outcomes. Inspection of the studentized residuals indicated that none of the studies had a value exceeding ±2.77, thereby eliminating the presence of outliers within this model. Cook’s distances further affirmed that none of the studies could be considered excessively influential. Neither the rank correlation nor the regression test revealed any asymmetry in the funnel plot (*P* = 0.119 and *P* = 0.062, respectively) (Fig. 4).

### Staged vs concurrent removal: meta-analysis

**Figure 5:**
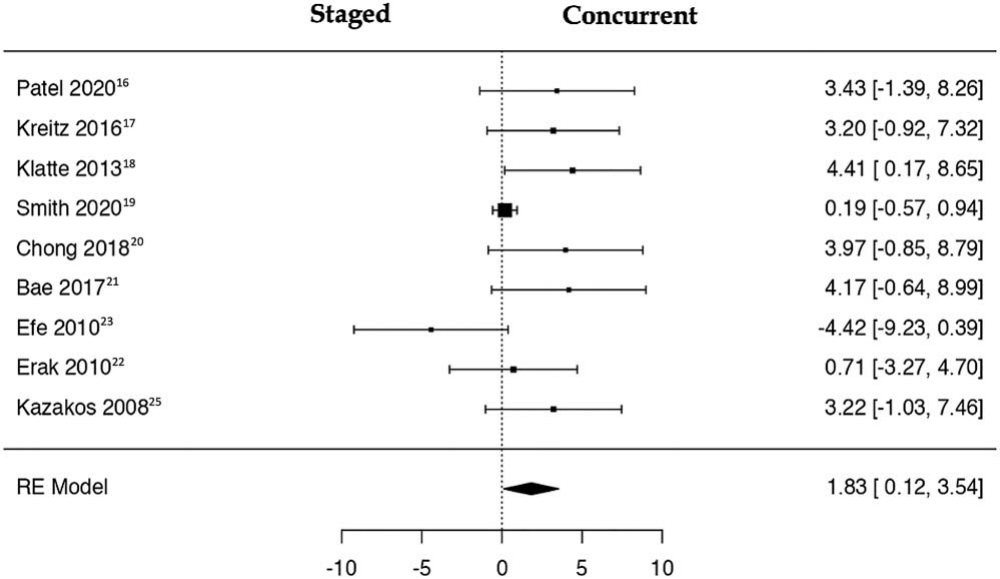
Risks of PJI in patients with previous hardware before total knee arthroplasty who underwent staged vs concurrent removal are depicted in the forest plot. The presented calculations were derived using random-effects models, and the confidence intervals are represented by the bars in the graph.

A total of nine studies (16, 17, 18, 19, 20, 21, 22, 23, 25) were included in the analysis, pooling data from 528 observations (patients with staged or concurrent hardware removal before TKA) and identifying 48 events (PJI after cTKA). Using the random-effects model, the data synthesis revealed a significant increase in the risk of PJI within the concurrent hardware removal group (log OR: 1.83, 95% CI: 0.15, 3.54), with a significant deviation from zero (*z* = 2.093, *P* = 0.036) (Table 2). A negative OR indicates that the experimental group (staged) has a lower risk compared to the control group (concurrent). The relative forest plot is depicted in Fig. 5.

**Figure 6:**
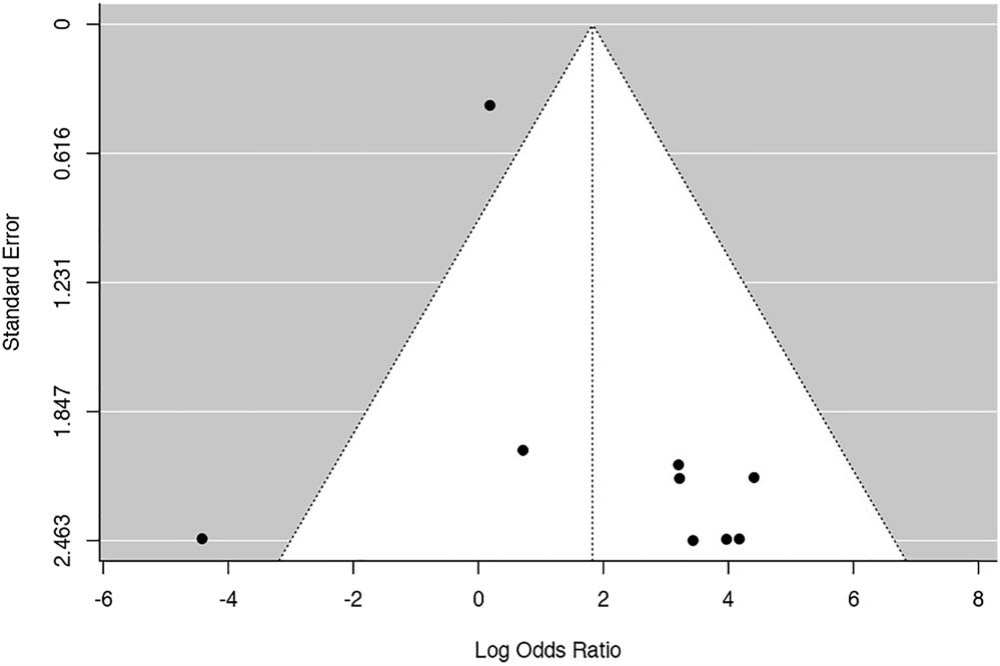
Funnel plot of the meta-analysis of staged vs concurrent removal: none of the studies could be considered excessively influential. Neither the rank correlation nor the regression test revealed any asymmetry in the funnel plot.

The *Q*-test results indicated heterogeneity in the true outcomes (*Q* = 16.757, *P* = 0.033, *τ*^2^ = 2.930, *I*^2^ = 50.5%). Inspection of the studentized residuals suggested that none of the studies had a value exceeding ±2.77, thus eliminating the presence of outliers within this model. Cook’s distances further affirmed that none of the studies could be considered excessively influential. Neither the rank correlation nor the regression test revealed any asymmetry in the funnel plot (*P* = 0.477 and *P* = 0.283, respectively) (Fig. 6).

### Meta-analysis of minor implant removal: staged vs concurrent

**Figure 7:**
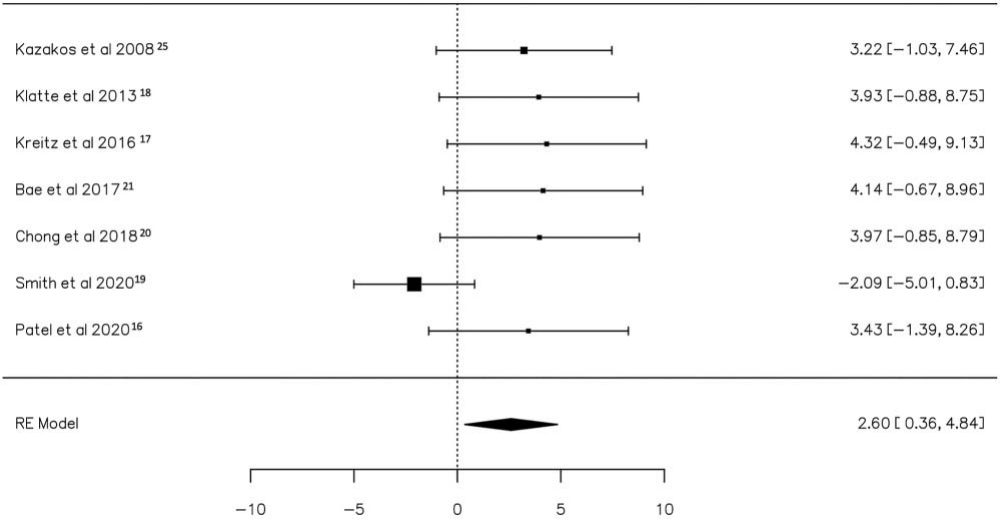
Risks of PJI in patients with implanted minor hardware before total knee arthroplasty who underwent staged vs concurrent removal are depicted in the forest plot. The presented calculations were derived using random-effects models, and the confidence intervals are represented by the bars in the graph.

A total of seven studies (16, 17, 18, 19, 20, 21, 25) were included in the analysis, pooling data from 216 observations (patients with staged or concurrent minor hardware removal before TKA) and identifying 15 events (PJI after cTKA). Using the random-effects model, the data synthesis revealed a significant increase in the risk of PJI within the staged hardware removal group (log OR: 2.60, 95% CI: 0.36, 4.84), with a significant deviation from zero (*z* = 2.274, *P* = 0.023) (Table 2). A positive OR indicates that the experimental group (staged) had a higher risk compared to the control group (concurrent). The relative forest plot is depicted in Fig. 7.

**Figure 8:**
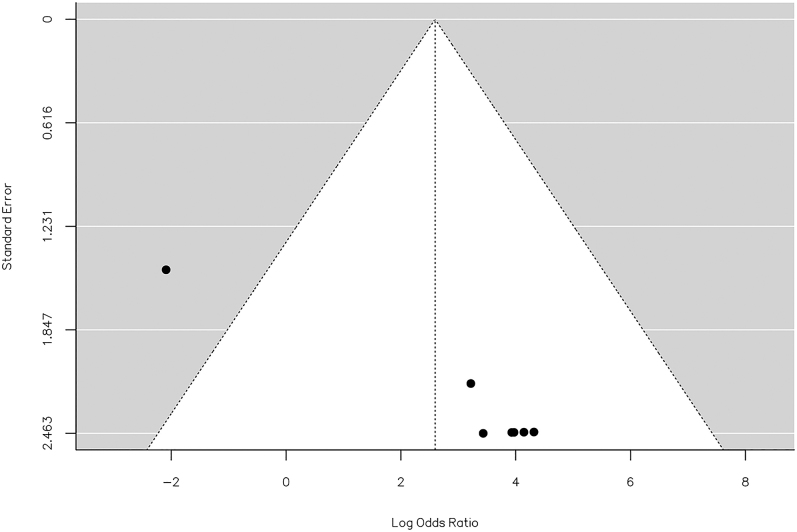
Funnel plot of the meta-analysis of minor hardware staged vs concurrent removal: one of the studies could be considered excessively influential. The regression test indicated funnel plot asymmetry (*P* = 0.0010) but not the rank correlation test (*P* = 1.0000).

The *Q*-test results indicated no significant heterogeneity in the true outcomes (*Q* = 11.104, *P* = 0.085, *τ*^2^ = 4.082, *I*^2^ = 45.7%). Inspection of the studentized residuals suggested that one of the studies had a value exceeding ±2.69, thus suggesting the presence of outliers within this model (19). Cook’s distances further affirmed that one of the studies could be considered excessively influential (19). The regression test indicated funnel plot asymmetry (*P* = 0.001) but not the rank correlation test (*P* = 1.000) (Fig. 8).

### Meta-analysis of major implant removal: staged vs concurrent

**Figure 9:**
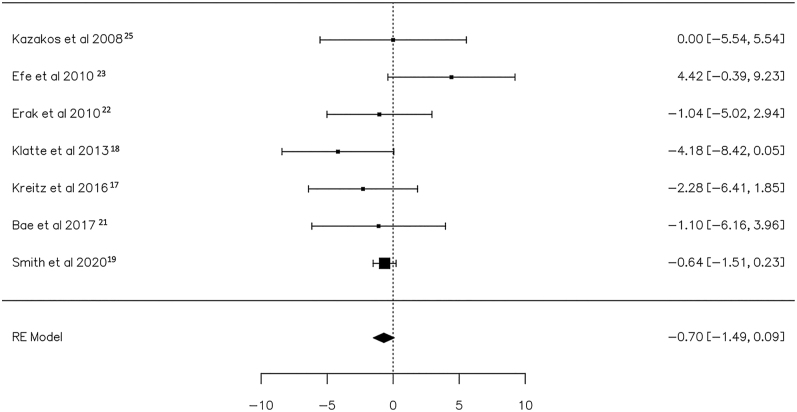
Risks of PJI in patients with implanted major hardware before total knee arthroplasty who underwent staged vs concurrent removal are depicted in the forest plot. The presented calculations were derived using random-effects models, and the confidence intervals are represented by the bars in the graph.

A total of seven studies (17, 18, 19, 21, 22, 23, 25) were included in the analysis, pooling data from 313 observations (patients with staged or concurrent major hardware removal before TKA) and identifying 33 events (PJI after cTKA). Using the random-effects model, the data synthesis revealed no significant differences in the risk of PJI (log OR: −0.70, 95% CI: −1.49, 0.09), with no significant deviation from zero (*z* = −1.748, *P* = 0.080) (Table 2). The relative forest plot is depicted in Fig. 9.

**Figure 10:**
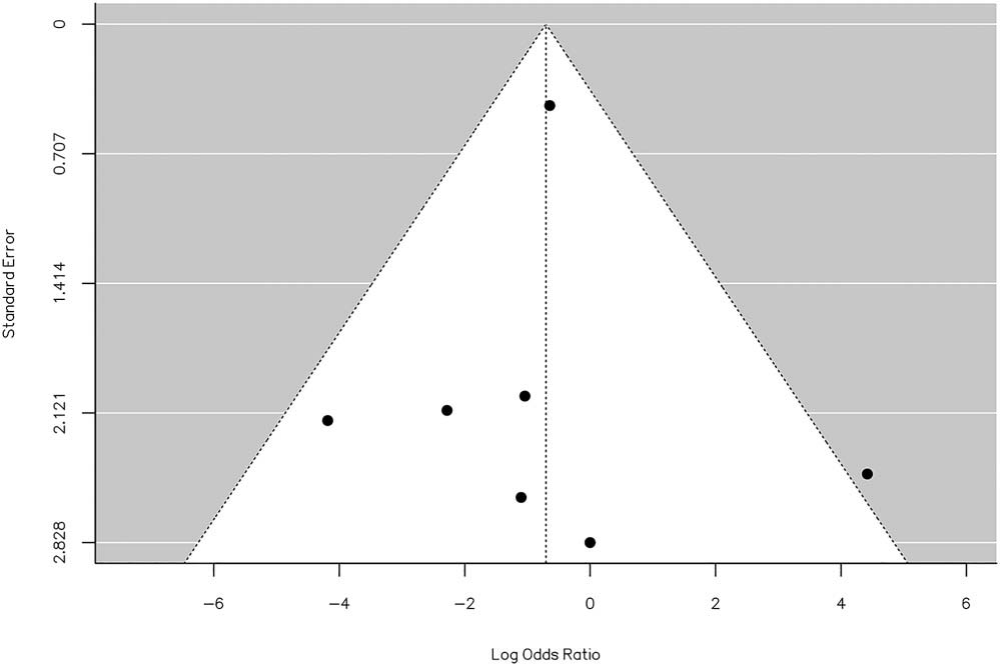
Funnel plot of the meta-analysis of major hardware staged vs concurrent removal: none of the studies could be considered excessively influential. Neither the rank correlation nor the regression test indicated any funnel plot asymmetry (*P* = 1.000 and *P* = 0.888, respectively).

The *Q*-test results indicated no significant heterogeneity in the true outcomes (*Q* = 7.639, *P* = 0.266, *τ*^2^ = 0.000, *I*^2^ = 0.00%). Inspection of the studentized residuals suggested that none of the studies had a value exceeding ±2.69, thus excluding the presence of outliers within this model. Cook’s distances further affirmed that none of the studies could be considered excessively influential. Neither the rank correlation nor the regression test indicated any funnel plot asymmetry (*P* = 1.000 and *P* = 0.888, respectively) (Fig. 10).

## Discussion

PJI in TKA following previous hardware fixation is currently a challenge for surgeons. As the number of patients requiring TKA continues to dramatically increase, surgeons can expect to see increasing number of patients with a history of previous surgery. Only few studies in the literature have focused on the infection rate following TKA surgery; therefore, we believe this is a highly relevant topic, useful for providing surgeons with important advice.

Periprosthetic infections remain the most feared complication after TKA. According to some authors, TKA in patients with previous knee surgery leads to an increased risk of infections (26, 27). The literature still does not provide a clear statement about the management of patients with previous knee implants undergoing TKA to reduce infectious complications (19, 28). The current literature presents several approaches regarding the removal of hardware before surgery: some studies suggest that staged removal, where hardware is removed in a separate procedure before TKA, may reduce infection risks compared to concurrent removal during TKA (6). However, other evidence indicates that either approach can lead to complications, including an increased risk of PJI when hardware is retained or removed improperly (19, 29). Individual patient risk factors and the surgeon’s clinical judgment often guide the decision. To our knowledge, this is the first systematic review in the literature with the aim to compare the infectious risk among patients with a previous hardware implantation undergoing TKA, meta-analyzing data to give solid evidence on how to manage conversion TKA. The stringent eligibility criteria led to the exclusion of many studies with incomplete or missing data and/or that did not fully meet the above-defined including criteria. Nevertheless, despite the limited number of included studies, it was still sufficient to yield statistically significant results (*P* < 0.05).

The study was designed to get a clear statement regarding the comparison between hardware removal and maintenance, and, if removal was deemed necessary, to evaluate the merits of staged vs concurrent removal. Our findings indicate a higher risk of infection associated with hardware removal compared to cases where the hardware was retained. Several contributing factors, including prolonged operative time, increased blood loss and extended exposure of the skin incision for hardware removal, play crucial roles in this outcome. Consequently, these considerations collectively influence the decision, when applicable, to retain the hardware, as per our study results.

According to Moussa *et al.*, (30) 53% of cases with concurrent hardware removal, tested positive for bacterial contamination in wound swabs, and in sonication. None of these patients was treated with antibiotics, and none developed deep infections. Then, they demonstrated that positive cultures obtained during hardware removal, in the absence of clinical signs of infection, are not meaningful. Similar results were obtained by Klatte *et al*. (18) who reported in their study only one case (<1%) of deep infection after single-stage hardware removal and following cTKA, after a mean follow-up of 5.4 years. The single patient who developed a late deep infection did not show bacterial growth in the preoperative and intraoperative samples.

Certain studies (20, 22) underscored the necessity of hardware removal for restricting the TKA procedure. When compelled, our study indicates a preference for a staged approach, revealing a higher OR for infection risk in cases of concurrent removal.

Nevertheless, in such scenarios, the adoption of new technologies proves beneficial. Specifically, the utilization of a navigation system for TKA can obviate the need for hardware removal (31). This technology not only facilitates precise implant positioning (32, 33) but also minimizes the extent of the bone cut (34) and preserves the intramedullary canal integrity, thereby preventing potential interference from hardware within the canal (35, 36).

Moreover, we also evaluated infectious outcomes in different types of implants (minor or major) and, therefore, analyzed the best removal strategies.

Our findings reveal distinct outcomes in infection rates between the two categories of procedures. For major surgeries, the meta-analysis did not show statistically significant differences in infection rates between the concurrent and staged removal groups. However, in the case of minor surgeries, the meta-analysis indicated a higher risk of infection associated with staged removal compared to concurrent removal. This suggests that, for minor hardware, a one-stage approach may be more advantageous in minimizing infection risk. This finding contrasts with general data but aligns with the specific nature of the procedures classified as ‘minor’. In these cases, the hardware is smaller, and its removal typically does not necessitate extended operative time or significant soft tissue disruption. As a result, the reduced surgical complexity and smaller implant size allow for a concurrent one-stage approach that effectively minimizes the risk of postoperative infections. The present study has certain limitations. First, only retrospective studies were identified, introducing a potential bias, due to lack of prospective studies. In addition, the study did not differentiate between hardware types and surgical procedures; therefore, the inclusion of both post-traumatic and elective surgeries, such as ACL reconstruction and HTO, without distinction, could impact the results. The use of unclear and varied diagnostic criteria to rule out periprosthetic joint infection likely contributed to the heterogeneity among studies and the pooling of results.

However, this study significantly contributes to the literature on this topic by offering valuable meta-analytic data that can aid surgeons in their daily practice when dealing with TKA in knees with previously implanted hardware. Furthermore, being the only existing meta-analysis on this subject in the literature, it stands as a valuable resource for the scientific community.

## Conclusions

Based on the data revealed in this meta-analysis, TKA following a previous knee hardware implantation indicates a higher risk of infection when the hardware is removed compared to leaving it in place. In cases where hardware removal is deemed necessary due to surgical requirements or space constraints, a staged removal is recommended. This approach is associated with a lower risk of periprosthetic joint infection compared to concurrent one-stage removal. The presence of minor hardware is the only scenario where, if removal is necessary, a concurrent one-stage approach is preferred due to the lower risk of infection compared to the two-stage approach.

## ICMJE Statement of Interest

The authors declare that there is no conflict of interest that could be perceived as prejudicing the impartiality of the work.

## Funding Statement

This work did not receive any specific grant from any funding agency in the public, commercial or not-for-profit sector.

## Author contribution statement

EF and CC screened the studies and extracted the data. DDM and TA evaluated the quality of the studies. DDM and CC wrote the manuscript. GB and MM corrected the whole paper. All authors read and approved the final manuscript.

## Data Availability

The datasets used and/or analyzed during the current study are available from the corresponding author on reasonable request.
